# Caregiver Views on Prospective Use of Robotic Care in Helping Children Adapt to Hospitalization

**DOI:** 10.3390/healthcare10101925

**Published:** 2022-09-30

**Authors:** Meiling Jin, Hanna Choi

**Affiliations:** 1 Research Institute of Nursing Science, Seoul National University, Seoul 08826, Korea; 2 Department of Nursing Science, Nambu University, Gwangju 62271, Korea

**Keywords:** child patient, care robot, caregiver, hospitalization

## Abstract

Children in hospitals endure a variety of stressful situations. Children feel friendly toward and have fun with robots. Care robots are considered to be an alternate technique to relieve stress after hospitalization. A mixed-methods study was conducted on caregivers to understand the ideal care robot. One hundred and fifty caregivers of pediatric patients participated in a quantitative online survey, and eleven participated in focus group interviews for qualitative analysis. Quantitative data underwent descriptive statistics. Content analysis was conducted for qualitative data. Regarding the overall awareness and necessity of a care robot, the caregivers thought it would help patients adapt to the hospital environment more quickly. The caregivers’ preferred character-shaped robots of child height. For sound, they preferred an animated character’s voice. For movement, they preferred the robot to roll on wheels. Regarding functions, medicine was the item for which they most wanted to use game elements. For the educational element, the caregivers wanted to teach children the reasons for and methods of medicine administration. Four themes were derived from the qualitative results. The findings are expected to contribute to the future development of care robots that can assist pediatric patients.

## 1. Introduction

Children in hospitals endure a variety of stressful situations, including physical discomfort and fear, which make it difficult for them to adjust to their new surroundings [1]. Pain from treatment, in which attention conversion therapy is an example of a pain reduction approach, is one of the most common pressures faced by pediatric patients [2].

Using robotics technology, care robots follow individual health goals and promote health [3]. Because children feel friendly towards and have fun with robots, care robots are considered to be an alternate technique to relieve stress after hospitalization [4]. The following are the different types of care robots that have been developed and utilized in hospitals for children.

The Probo robot was created to provide knowledge and spiritual support to children, as well as to console children undergoing treatment [5]. The MediRobbi robot is an interactive robot designed to assist and guide medical procedures [6]. The Nao robot is a humanoid robot that relieves children’s physical discomfort during flu vaccines [7]. Pleo is a dinosaur robot that helps children have a cheerful attitude while fighting disease, which enhances therapy effectiveness and the well-being of hospitalized children [8,9]. A bear-shaped, huggable robot was made to help children with stress, worry, and pain [10]. The Arash robot was created to help children cope with the discomfort of cancer treatment by entertaining and supporting them [11]. The Pepper robot helps children learn in the home environment, serve as a companion to older adults, and coach elderly people with psychiatric disorders through rehabilitation and recreational activities [12]. The above robot studies mainly targeted children with autism spectrum disorder, and most of them focused on the emotional aspects of children, except for the Pepper robot. According to the literature review, previous studies have focused on evaluating children’s distraction potential during medical procedures using robots, as well as the emotional support and well-being of children during hospitalization [13]. Therefore, this study tried to develop a new robot that helps with children’s general hospitalization in detail. We determined at which age they most need robots, where to place them, what functions the robots should have, and what role the robot should play.

For understanding the potential use before development, researchers have identified care robots’ proactive role in children’s hospitals from the nurse’s point of view through interviews [14]. There are many limitations to the ability of health care providers to understand the full needs of children with limited communication skills.

The system-development life-cycle methodology is used in the development of new technologies. It consists of four steps: needs analysis, design, implementation, and evaluation [15]. Needs analysis is the first step in this process. We intended to analyze the need for robots that help children adapt to hospital life through the use of caregivers who take care of the children closest to them.

This study intended to provide basic data for the development of care robots that help children adapt to hospitalization by examining the need for robots. However, toddlers are limited in their ability to express themselves freely and have limitations due to their lack of understanding of terms. According to the research conducted so far, children are used to playing with robots that have already been developed and analyzed through very simple yes or no questions [16]. Moreover, in Korea, when a child is hospitalized, it is the cultural norm that their caregiver stays in the room with them and looks after them, so parents are effective in verbal and nonverbal communication with the child [17]. Therefore, it is meaningful to investigate and analyze the needs of the caregivers who understand them best because they care for the children closest to them.

Scholars have pointed out that it is necessary to use different methods of data collection or analysis according to the content, object, and purpose to be grasped through demand analysis. In existing papers, most of the statistical processing uses questionnaires, so it is necessary to use a mixture of two or more methods to find a complementary relationship, rather than using one method [18]. For effective data collection, it is necessary to use two or more methods (e.g., questionnaire and interview) [19].

The mixed research method, which combines both methods, is a disadvantage of quantitative research, in that it is difficult to understand the research subjects’ situational context, and it is difficult for direct opinions and statements to reflect the research data. The disadvantage of qualitative research is researcher bias. As a research method that overcomes the excessive reflection of this problem or the difficulty in generalizing research results, it has the advantage of complementing the shortcomings of the two research methods [20].

While building a care robot, it is critical to understand what kind of robot children require from the perspective of the caregivers who are closest to them. From the perspective of caregivers, the goal of this study was to suggest the appearance, function, and role of a care robot that could be deployed in children’s hospitals.

## 2. Materials and Methods

### 2.1. Study Design

This mixed-methods study included caregiver questionnaires and focus group interviews with children aged 3 to 18 who had been hospitalized in the previous 3 months. The research questions were as follows:

- Quantitative Question 1: Do you think care robots are needed in children’s hospitals?
- Quantitative Question 2: What do you think the appearance of the care robot should be for hospitalized children?
- Quantitative Question 3: What do you think is the most necessary function of a care robot for hospitalized children?
- Quantitative Question 4: Where would be the best place to locate the care robot?
- Qualitative Question 5: What do you think a care robot’s role should be for hospitalized children?

### 2.2. Participants

The participants in this study were caregivers of children aged 3 to 18 who had been hospitalized for at least 3 days within the previous 3 months. Caregivers of children with chronic diseases, cancer, or in need of intensive care were excluded from the study. All participants filled in consent forms for participation in the study. 

### 2.3. Data Collection

The online survey for caregivers of hospitalized children was commissioned by Korea Research, a specialized research institute, and the survey period was from 16 March to 21 March 2020. The researcher sent the research participant selection criteria, research participant recruitment letter, research participant explanation and consent form, and questionnaire to the academic research officer of Korea Research, and then the person in charge produced it according to the survey frame of Korea Research. The researcher then confirmed it. In addition, Korea Research established an online survey web link for to be sent to the survey subjects for testing, and the researcher launched the survey after checking the contents of the web link. The subjects of the survey read the research participant recruitment statement and the research participant explanation presented through the online survey web link from Korea Research. If they did not click a button indicating they agreed to participate, then the survey automatically closed and the session ended. We did not use demonstration programs or videos to investigate the need to develop a robot that helps children adapt to hospitalization. If the caregiver saw or experienced a video or demonstration of a robot that was already developed, they may have preconceived thoughts about it. We recognized that these are suitable for usability evaluation research. For the qualitative focus group, interviewees were recruited through Korea Research, and an online survey was conducted from 16–21 March 2020. On 31 January 2020, the researcher divided 11 participants into two groups and interviewed them for about 60–90 min each in the seminar room of Seoul National University’s College of Nursing; all contents were recorded.

Participants were also asked seven open-ended questions about the experience of their children’s hospitalization and the need for and role of a care robot (Table 1).

### 2.4. Data Analysis

The results of the online survey were analyzed using SPSS 23.0 for Windows. The percentages, means, and standard deviations were calculated according to the survey items for each field of the questionnaire. Interview data recorded in the focus group interviews were transcribed and analyzed in the language used to establish the reliability of the data. Qualitative content analysis was used to analyze the collected interview data. 

Qualitative content analysis is a research method that analyzes the patterns and topics of content through a coding process and a systematic classification method based on the overall understanding of vast textual data, such as interview data [21]. Without using a software program, two researchers analyzed the content in the following way. The specific analytical steps were as follows: First, the researcher repeatedly read the transcription and tried to understand it. Second, the representative main concepts and contexts were coded in the overall content. Third, if commonalities were found between the extracted codes, they were grouped separately and classified into topics. 

### 2.5. Verifying the Validity of the Research Results

After revising a questionnaire developed by the researcher for nurses to suit the purpose of this study, four experts modified it to ensure content validity [22]. The experts consisted of one person enrolled in a PhD program in child nursing, one with a PhD in child nursing, and two professors of nursing informatics. Each expert calculated the content validity score for each item, assigning 4 points if it was valid, 3 if it was somewhat valid, 2 if it was somewhat invalid, and 1 if it was not valid at all. We then calculated the content validity index for each item and guided the experts to present their opinions freely. Three items with a content validity index of 0.8 or less, and those with ambiguous meanings, were excluded. As a result, quantitative data were used to define seven questions on general characteristics, seven on overall awareness and necessity of care robot, four on the appearance of care robots, two on the function of care robots (one on game elements and one on educational elements), and one on the place for a care robot.

As for qualitative data, findings of the care robot role were verified according to credibility, auditability, fittingness, and confirmability suggested by Sandelowski [23]. For the credibility of the data, subjects were recruited using the random sampling method from a Korean company that conducts research. To ensure auditability, we recorded all processes of the actual steps from the planning to the reassurance of all study findings from the participants. Six professionals who were experts in their fields (three nursing researchers, two health researchers, and one qualitative researcher) refined the final contents for testing fittingness and confirmability. As for the validity of the qualitative results, we asked each focus group the same set of questions. 

## 3. Results

### 3.1. Quantitative Result

#### 3.1.1. General Characteristics

A total of 150 participants took the online survey for quantitative data collection. The online survey of caregivers drew a total of 63 men and 87 women. Regarding age, 47.3% were in their thirties, and most of the caregivers had school-aged children. The average age of the participants was 40 ± 5.9 years. The average length of stay in the hospital was 6 days, ranging from 3 to 26 days, and many children were primarily hospitalized for pneumonia (Table 2).

#### 3.1.2. The Overall Awareness and Necessity of Care Robots

The participants responded based on a five-point scale (1—definitely no, 2—no, 3—yes, 4—definitely yes, 5—neither). The scores for each category were all over 3 when it came to general perceptions and the requirement of a care robot. “A care robot is needed in pediatric wards” scored 3.41 ± 0.94; “A care robot will reduce the fear of hospitalization” scored 3.33 ± 0.82; “A care robot will assist child patients to adapt to the hospital environment more quickly” scored 3.33 ± 0.73; “A care robot will enhance child patients’ cooperation with treatment” scored 3.37 ± 0.71; “A care robot will reduce the fear of treatment” scored 3.32 ± 0.73; and “A care robot will ease the burdens of parents looking after their children” scored 3.25 ± 0.77. 

Kindergarteners (37.9%) were the age group most in need of a care robot according to the caregivers. Daycare was 26.4%, lower elementary school was 22%, upper elementary school was 5.1%, middle school was 5.4%, and high school was 3.2%. The caregivers thought children in kindergarten were the right age to benefit from a care robot, claiming that children would like to have one. They also stated that children in hospitals become frustrated and bored, but that if they had a care robot, they could be friends with it. Hospital beds, according to the caregivers, are the places where care robots are most needed.

#### 3.1.3. The Appearance of Care Robots

Character-shaped robots were the most popular among the caregivers (including animation characters). When asked why they preferred character-based robots (including animated characters), the caregivers said it was because they seemed comforting and nice to the children.

When asked what size the care robot should be, 42.3% said it should be the same height as the young patients; 46.5% indicated an animated character’s voice would be preferred for the care robot’s sound. In terms of the way the care robot should move, 56.9% thought it should be on wheels (Table 3).

#### 3.1.4. The Function and Place of a Care Robot

When asked if gaming elements relating to nursing services should be utilized to encourage taking medicine, 32.3% of the caregivers said yes. The caregivers’ arguments for choosing this item were that they would enjoy it if children were encouraged as if they were being rewarded for taking medicine, and that children would be more willing to take medicine if they were made to feel better through games (Table 4).

When asked what information they would like their pediatric patients to learn via a care robot, 20.7% of the caregivers said the reason for and method of medicine administration. When asked why they chose this item, the caregivers replied that children would listen better if the care robot explained the purpose for medicine administration (Table 4).

The caregivers of hospitalized children accounted the hospital bed to be the place where a care robot was needed 30% of the time. This was followed by the treatment room (25.5%), hospital corridor (20.1%), nurse station (9.9%), EMR (electronic medical record) cart (9.6%), and school in hospital (4.8%).

### 3.2. Qualitative Result

#### 3.2.1. General Characteristics

We interviewed eleven participants in the focus group for the qualitative research. The caregivers who took part in the focus group interviews were all full-time, stay-at-home mothers, with an average age of 41 ± 4.2. The children were divided into six girls and five boys, with an average age of 9 years and a 6-day hospitalization. The most common reason for hospitalization was pneumonia (Table 5).

#### 3.2.2. The Role of a Care Robot

The following are the outcomes of the focus group interviews, which provide the perspectives of both hospitalized children and their caregivers (Table 6).

#### 3.2.3. Children’s Perspective

- Relieving fear by providing familiar elements

According to the caregivers, hospitalization is a scary event for children. The caregivers claimed that the kind expression of a care robot could help decrease fear in children on the day of their admission. Children would prefer it if the familiarly characterized care robot complimented, cheered, or encouraged them when they had to do tasks they disliked, such as taking medicine or traveling to the treatment room.

*“I believe that a care robot would be ideal for removing the fear of being admitted to the hospital for the first time…. He was unwell and sensitive, but he was abruptly placed in an unknown atmosphere, so the odd devices and injections were terrifying to him. I believe the care robot will be beneficial to youngsters”*

(Participant 5, Focus Group 1).

*“You had a rough day today, right?’ the care robot asked. ‘Isn’t it excruciating? Let us, on the other hand, take good care of ourselves today.’ … I felt [the care robot] would be able to help people cope with their fear”*

(Participant 1, Focus Group 1).

- Expecting a care robot to be a friend

The caregivers expressed their desire for a care robot to be a friend who speaks to and plays with the child patients. Patients admitted to the hospital said that boredom was one of the biggest difficulties they faced because they spent most of their time watching YouTube without any activity. In particular, the unfamiliar environment maximizes the fear of the first day of hospitalization. At this time, friends other than family could alleviate this fear. Therefore, they hoped care robots could be friends who talk to or connect with patients.

*“I hope the care robot is like a friend. My child is anxious, so when I go to restroom, she worries and tells me to come right away, even child is left alone for a short time. At times like this, a robot can communicate when a child asks something. If the care robot with no reaction, I think it’s not much different from tablet PC.”*

(Participant 2, Focus Group 2).

*“Some children have cell phones, but many do not. I think it would be good to use the [robot’s] Wi-Fi function to make video calls possible and connect to talk with or see the faces of their friends or mother.”*

(Participant 4, Focus Group 2).

- Educating tool

Children are fascinated by care robots, so if the robots teach them lessons to which they ordinarily do not listen (e.g., hand cleaning or respiratory therapy), they are likely to accept them. The caregivers also suggested that a care robot could explain to children why they should do tasks they dislike, such as taking medicine or receiving injections. Furthermore, in this scenario, children may forget information shortly after learning it; therefore, it would be beneficial to reinforce it is using a care robot. The caregivers expressed their desire for the care robot to assist children in navigating an unfamiliar environment when they were admitted to the hospital.

*“I’d like a care robot to explain disease to the child in a timely manner at a level the child could understand. “Could a child be taught to wash his or her hands if the lesson were presented as a children’s animation? The surgery will proceed in this manner. Therefore, don’t worry when you’re done, do something with me.”*

(Participant 5, Focus Group 1).

#### 3.2.4. Caregivers’ Perspective

- Relieving stress to provide a little break

One caregiver said she could not sleep because she had to care for her child 24 h a day and that it was very difficult to feed their child and administer medicine to them when the child was hospitalized. Furthermore, the caregiver stated that she struggled physically and mentally when her child was unwell, cranky, or upset. In this scenario, the caregiver stated that she would be able to rest briefly if the care robot talked to or played with the child.

*“The mother can take a break while the child converses with the robot. As a result, I believe it will benefit me emotionally because I will be able to take a break.”*

(Participant 5, Focus Group 1).

*“If [the care robot] plays with their child for even an hour, I believe it will be lot less stressful and more pleasant for caregivers.”*

(Participant 2, Focus Group 2).

### 3.3. Suggestion for the Adaption of a Care Robot for Children’s Hospitalization

From the perspective of the caregivers, the installation location, the care robot’s appearance, the type of care robot that hospitalized children are most likely to prefer, and the care robot’s implementation function are proposed as follows (Table 7). 

## 4. Discussion

### 4.1. Overall Awareness and Necessity of a Care Robot

Regarding the overall perception of and necessity for care robots, in the category of “Using care robots, the burden on parents caring for hospitalized children will be reduced”, the caregivers of hospitalized children scored high. This result was also consistent with the theme of “Relieving stress to provide a little break”; therefore, it is judged that care robots are expected to relieve the stress of care.

The caregivers of hospitalized children said that preschool age is the age at which care robots are most needed. Preschool is a time when peers are important, and children can play with friends using rules [24]. Moreover, during this stage, they begin to feel interested in playing with their friends. Based on these characteristics, it is said that if a care robot becomes a friend and plays with a pediatric patient, it will help the child’s adaptation to hospitalization life by aiding cognitive and emotional development by accepting roles and resolving realistic frustration.

### 4.2. The Care Robot Appearance

From the standpoint of the caregivers, a cartoon character-based robot was the favored type of care robot. A character-based robot was also the favored form of care robot in a study of pediatric nurses [22]. This result was the same as another study of children aged 4–7 years [25]. The preferred size was discovered to be around the same height as the child in this investigation. 

Other research has revealed that children prefer smaller robots to larger robots [26]. When it came to the movement of the care robot, the caregivers favored it rolling on wheels. According to previous surveys, nurses and doctors preferred that the robot move on wheels (71.1%) [27]. The robot’s movement, according to Jin and Kim [22], preferred walking shape. The researchers believe that rolling with wheels is more stable and does not hurt patients. As a result, we believe it is important to develop a care robot around these requirements. According to Arnold et al. [26], when designing products for children, their opinions need to be gathered if they are to be considered. Therefore, young patients should be included in the design of care robots.

### 4.3. The Care Robot Function

The caregivers preferred medication in the game elements to provide a reason for and method of medicine administration in an educational manner via a care robot. Medication was the second-most prevalent item that might be implemented when utilizing game features according to pediatric nurses [22]. Baek et al. [28] found that, among children aged 6 to 12, 32.6% of children owned a smartphone and used game functions the most.

For children, games are a very familiar tool. Therefore, it is believed that if children experience taking medicine as a serious game, they will be more willing to actively participate in treatment. The other recommendations in the survey included discharge education, type of inspection, result of inspection, outpatient treatment, medication precautions, bedsore education, hospital tours (location), fall prevention education, explanation of surgery, education before and after surgery (deep breathing, fasting, and position), wound dressing, nursing method according to disease, and growth and development (characteristics of each age group). 

Robots that employ game elements have an emotionally supportive effect on children. Pourteimour and Kazemi [29] showed that, when preschool children were hospitalized, a robotic game kit could be used to lower the children’s separation anxiety and fears of physical injury. 

### 4.4. The Role and Place of a Care Robot

The caregivers anticipated that their children would be friends with a care robot. They wanted a robot that could converse with children, as well as encourage and play with them. The most significant responsibilities of a care robot are to bring comfort to inpatients, reducing anxiety, discomfort, and suffering, while enhancing the motivation to be treated and raising attentiveness [30]. Liang et al. [14] reported that nurses thought care robots were efficient and effective in providing hospital guidance for hospitalized children and their guardians, and that care robots programmed for education provided education for patients in the absence of nurses. Nurses thought robots could play a role in complementing the reinforcement of a patient’s knowledge and their self-management ability.

### 4.5. Limitations

This study investigated the necessity for a care robot that can assist children in adjusting to hospitalization. However, children were not included in this study due to being unable to identify differences in need due to these variables. Future research should consider various factors related to the characteristics of various age groups, disease severity, and disease type.

## 5. Conclusions

In this study, we used a mixed research method with the caregivers of hospitalized children so that we could understand the needs for the development of care robots regarding children’s adaptation to hospitalized life. As a result of this study, we discovered that the preferred care robot for children’s adaptation to hospitalization was a character robot with the voice of an animated character, a height similar to that of a hospitalized child, and wheels for movement. For developing a care robot, we required software that selects a child’s favorite animated cartoon character, hardware that utilizes a face-shaped monitor representing the selected character and possesses the capability of height adjustment. In addition, we found that there was a high demand for education on the reasons for and methods of medicine administration by adding an element of fun through serious games. The caregivers hoped that a care robot could relieve fear by providing familiar elements, serving as a friend and an educational tool for hospitalized children, and relieving stress in order to provide a little break to caregivers.

Based on the findings of this study, it is envisaged that care robots that assist hospitalized pediatric patients will be produced in the future. 

## Figures and Tables

**Table 1:** Guiding Questions for the Focus Groups.

Type	Question Topics
Introduction	Share the sociodemographic characteristics (gender, age, child’s age, child’s diagnosis, period of hospitalization)
Opening	During hospitalization, share your daily routine with your child What was the most memorable challenge you faced while in the hospital?
Exploratory	Do you think care robots are helpful to your child during hospitalization? Could you recommend a care robot’s functions that would help with the difficulties of hospitalization? Please describe in detail the role of the robot you expect in the ward.
Closing	Share a summary of the discussed content. (After summarization) Was it well summarized? Do you have any comments you would like to add?

**Table 2:** General Characteristics of the Caregivers in the Quantitative Analysis (*n* = 150).

Characteristics		*n* (%)
Gender	Male	63 (42)
	Female	87 (58)
Age	20 s	2 (1.3)
	30 s	71 (47.3)
	40 s	66 (44)
	≥50 s	11 (7.3)
	Mean ± SD = 40 ± 5.9	
Education	High school	14 (9.3)
	College	16 (10.7)
	University	101 (67.3)
	≥Graduate school	19 (12.7)
Job	Homemaker	39 (26.0)
	Office clerk	64 (42.7)
	Tradesperson	3 (2.0)
	Professional	26 (17.3)
	Public official	6 (4.0)
	Private business	10 (6.7)
	Other	2 (1.3)
Hospitalization Experience	Currently hospitalized for 3 days or more	16 (10.7)
	Have been hospitalized within the last 3 months	134 (89.3)
Child’ age	Preschool	50 (33.3)
	School	63 (42)
	Adolescence	37 (24.7)
Child’s diagnosis	Pneumonia	35 (23.3)
	Enteritis	25 (16.7)
	Fracture	22 (14.7)
	Influenza	21 (14.0)
	Other	47 (31.3)

**Table 3:** Care Robot Appearance: Choosing Children’s Preferred Robot Characteristics from Caregivers’ Perspectives (Multiple Choice).

Type	Ranking	Item	*n* (%)
Design	1	Character-shaped robot (including animated characters)	138 (45.5)
	2	Animal-shaped robot	108 (35.6)
	3	Humanoid robot	35 (11.6)
	4	Other (monitor-shaped robot, etc.)	22 (7.3)
Size	1	The height of the pediatric patients	90 (42.3)
	2	Taller than the height of the pediatric patients	52 (24.4)
	3	Smaller than the height of pediatric patients	36 (16.9)
	4	Pediatric patient is sitting on a bed	28 (13.1)
	5	The height of an adult	7 (3.3)
Sound	1	Animated character voice	128 (46.5)
	2	Familiar to pediatric patients	83 (30.2)
	3	A sound that expresses various types of music	37 (13.5)
	4	Sound like a real animal	27 (9.8)
Movement	1	Rolls on wheels	111 (56.9)
	2	Walking	67 (34.4)
	3	Fixed form	17 (8.7)

**Table 4:** Care Robot Function (Multiple Choice).

Element	Ranking	Item	*n* (%)
Game (Items to be implemented using game elements in nursing procedures)	1	Medication	103 (32.3)
	2	Injections	90 (28.2)
	3	Measure blood pressure, body temperature, and pulse	44 (13.8)
	4	Food rejection	42 (13.2)
	5	Diet survey	19 (6.0)
	6	Inspection	11 (3.4)
	7	Changing clothing in hospital	10 (3.1)
Educational (Topics to be taught to the children through care robot)	1	Reason for and method of medicine administration	68 (20.7)
	2	Overall description of the disease	48 (14.6)
	3	Reason for and method of inspection	42 (12.8)
	4	Infection management education	40 (12.2)
	5	Inpatient education	21 (6.4)
	6	Other (discharge education, type of inspection, result of inspection, etc.)	110 (33.4)

**Table 5:** General Characteristics of the Caregivers in the Qualitative Analysis (*n* = 11).

Number	Gender	Age	Period of Hospitalization	Child’ Gender	Child’ Age	Child’s Diagnosis
1	F	31	5	F	3	Bronchopneumonia
2	F	40	6	M	3	Bronchopneumonia
3	F	43	4	M	9	Supernumerary Teeth
4	F	44	9	F	12	Mycoplasma Pneumonia
5	F	46	4	M	14	Acute appendicitis
6	F	39	5	F	7	Pneumonia
7	F	45	4	M	14	Fracture
8	F	44	9	F	6	Obstructive pulmonary Disease
9	F	39	10	F	10	Pneumonia
10	F	42	8	M	15	Accessory Navicular Syndrome
11	F	39	6	F	6	Pneumonia

**Table 6:** The role of care robot in children’s hospital.

Domain	Sub-Domain	Quote
Children’s perspective	Relieving fear by providing familiar elements	*I believe that a care robot would be ideal for removing the fear of being admitted to the hospital for the first time*
	Expecting that care robot can be a friend	*I hope the care robot is like a friend. My child is anxious, (..) even child is left alone for a short time. A robot can communicate when a child asks something*
	Educating tool	*I’d like a care robot to explain the disease to the child in a timely manner*
Caregivers’ perspective	Relieving stress to provide a little break	*If [the care robot] plays with their child for even an hour, I believe it will be lot less stressful and more pleasant for caregivers*

**Table 7:** Suggestion for the adaption of a care robot for children’s hospitalization.

Category	Sub-Category		Detail
Main target population	3–10 years old (daycare, kindergarten, lower elementary school)		
Care robot location	Hospital bedside		
Care robot’s appearance	Design	Character-shaped robot (including animated characters)	A round monitor in the face part allows children to set their favorite cartoon character on their own.
	Size	The pediatric patient’s height	A height adjuster is installed on the robot’s leg so that the height can be adjusted based on the child’s height.
	Sound	Animated character voice	Children can choose from kindergarten and lower elementary characters and franchises, such as Pororo and Marvel.
	Movement	Rolls on wheels	Wheel-based legs are utilized, as on the Pepper robot.
Care robot’s function	Game element	Medication	The care robot functions as a serious game. A child administers medicine to a character. Every time the character administers medicine, the child’s health level will increase. The child will receive coins, badges, etc., each time they take medication or undergo a medical procedure.
		Injections	
		Measure blood pressure, body temperature, and pulse	
		Food rejection	
	Educational element	Reason for and method of medicine administration	The care robot functions as a serious game. The child receives training from the chosen character through a face-shaped monitor. If the child listens carefully to the educational content and solves a problem, they will receive a coin or badge.
		Overall description of the disease	
		Reason for and method of inspection	
		Infection-management education	

## Data Availability

Data are available on request due to restrictions. The data presented in this study are available on request from the corresponding author.
